# Impact of Active Breaks in the Classroom on Mathematical Performance and Attention in Elementary School Children

**DOI:** 10.3390/healthcare9121689

**Published:** 2021-12-07

**Authors:** Giovanni Fiorilli, Andrea Buonsenso, Giulia Di Martino, Claudia Crova, Marco Centorbi, Elisa Grazioli, Eliana Tranchita, Claudia Cerulli, Federico Quinzi, Giuseppe Calcagno, Attilio Parisi, Alessandra di Cagno

**Affiliations:** 1 Department of Medicine and Health Sciences, University of Molise, 86100 Campobasso, Italy; fiorilli@unimol.it (G.F.); andreabuonsenso@gmail.com (A.B.); giulia.dimartino21@gmail.com (G.D.M.); marco.centorbi@hotmail.it (M.C.); giuseppe.calcagno@unimol.it (G.C.); 2 Department of Motor, Human and Health Sciences, University of Rome “Foro Italico”, 00197 Rome, Italy; claudia.crova@uniroma4.it (C.C.); elisa.grazioli@uniroma4.it (E.G.); eliana.tranchita@gmail.com (E.T.); claudia.cerulli@uniroma4.it (C.C.); fquinzi@libero.it (F.Q.); alessandra.dicagno@uniroma4.it (A.d.C.)

**Keywords:** children, physical activity, school time, academic achievement

## Abstract

Background: The increasing need to face the problem of sedentarism, especially in the COVID-19 era, induced teachers and researchers to find new intervention methodologies in school context. Active breaks (ABs) include brief periods of physical activity as a part of the curriculum. This study aimed to investigate the AB acute responses on attentive skills and mathematical performance and attention in a primary school. Methods: A total of 141 children (aged 9.61 ± 0.82), divided into six classes, participated in this study. Each class was randomly assigned to three groups on the basis of the type of protocol performed during the three ABs scheduled in a school day: fitness (FIT), creativity (CREAT), and control group (CON). At baseline and at the end of interventions, all participants underwent the Stroop Color and Word test (SCWT) and the math test (MATH) to assess the level of attention and mathematical performance, respectively. The degree of enjoyment was evaluated through the modified Physical Activity Enjoyment Scale. Results: The factorial ANOVA showed significant differences between the FIT and CON in MATH test (*p* = 0.023) and SCWT (*p* = 0.034). CREAT and FIT groups showed higher degree of enjoyment than the CON (both *p*s < 0.001). Conclusions: This study showed a positive acute impact of AB interventions. FIT positively influenced attentive and math performances more than the CREAT, probably due to the correct work/rest ratio and executive rhythm that allowed children to reach a good level of exertion. This report showed that ABs can be a useful and productive activity to be performed between curricular lessons.

## 1. Introduction

Nowadays, in primary schools, the time allocated for physical activity (PA) has been considerably lowered in favor of additional seated time for other academic tasks [1]. Considering the amount of time spent by children at school, school itself could be an ideal environment for promoting active lifestyles and reducing sedentary time. In this manner, the schools can guarantee these benefits to children, regardless of age, ethnicity, gender, and socio-economic status [2].

In addition, the COVID-19 pandemic, with its access restrictions to public recreational spaces and sportive areas, limited the possibility to keep children active. Consequently, a great number of children worldwide spent their daily activity at home with little access to structured activities, leaving schools to be the last bastion where PA is achievable. Sedentary attitude, due to lockdown, leads to adverse effects on children, such as mental swings, depression, and an impairment of the general health [3].

Epidemiological evidence has suggested that a sedentary lifestyle leads to an increasing risk of several clinical conditions [4] such as overweight [5] and cardiorespiratory impairments [6,7]. Moreover, an additional PA practice leads to better coordination and cognitive functions [8,9]. The link between PA and cognition is supported by evidence that cognitive and motor tasks activate the same neural regions [10,11]. The overlap in the brain areas, responding to both motor and cognitive processes, may be the mechanisms by which, during PA, the involvement of prefrontal structures and arousal in the brain tissue were observed [12].

Moreover, Diamond and Lee [13] suggested that enjoyment and interest in the performed activities are the principal factors to promote executive functions (EFs), school outcomes, and children’s emotional and social development.

The increasing need to face the problem of sedentarism and at the same time to enhance children’s cognitive development led teachers and researchers to find new useful methodologies in the school context. Active breaks (ABs) represent an alternative moment of the lesson aimed to include brief periods of PA as a part of the curriculum [14,15]. Watson and colleagues showed three ways of ABs administration: the interval between two consecutive lessons, breaks conducted during the lesson or physically active lessons, and the integration of PA into other disciplinary subjects [16].

Several authors [17,18,19,20] showed how acute active bouts of PA during the breaks between periods of academic instruction influence students’ on-task behavior. The acute effects include short moderate-to-vigorous intensity bouts of PA, increasing plasma catecholamines, improving activation and arousal, or restoring cognitive resources after challenging lessons [21]. Acute ABs promote an increase in time spent on tasks, reduction in distraction [22], and enhancement in the enjoyment of learning [23], with improvement of academic performance and cognitive functions [16,24,25].

Duration, intensity, and a specific type of physical exercise (cardiovascular or resistance exercises, with or without cognitive engagement) during ABs interventions leads to different outcomes [2]. Moreover, exercise with music represents a fundamental tool in this context, which positively influences affective arousal, mood, and emotional regulation [26]. Music induces the release of endorphin as well as inhibiting corticotrophins with a decrease of subjective fatigue [27] and solicits imagery and creativity with significant effects on EFs [28].

Currently, there have been no previous recorded active break study protocols proposed on creativity models to stimulate cognitive development. In order to perform the creative protocols, children need an efficient mental engagement by which they recognize stimuli and new information to master their skills suitable for the specific situations required [29]. The hypothesis of the correlation between high levels of creativity and academic achievement in students and the role that creativity has in academic performance improvement was confirmed by several studies [30,31,32]. The characteristics relevant in creative ability, such as flexibility and original thinking stimulating problem-solving strategies, play a fundamental role in the learning process [30].

Finally, enjoyment and interest really stimulate divergent solutions in math problems [33], promoting a better retention of information and developing easier learning of skills [34].

The present study aimed to investigate the acute responses of ABs and on task classroom behaviors (cognitive and attentive engagement) between periods of academic instruction in a primary school. A secondary aim was to identify the efficacy of ABs on the basis of creativity tasks, such as a new methodology suitable to reach the purposes mentioned above.

## 2. Materials and Methods

### 2.1. Study Design

A mixed 3 × 2 between-subjects experimental design compared two experimental and a control condition, measured at two time points (at baseline and post-test) to assess attentive and academic performances in primary school children. Baseline assessment data were collected in the week before the beginning of the intervention. At the end of the intervention, the same procedures were used to assess post-test results, starting with the attention test and successively the academic performance assessment. All the interventions were conducted during the normal class time by trained researchers blinded to the experimental condition.

### 2.2. Participants

One hundred forty-one children (aged 9.61 ± 0.82) of grades 3, 4, and 5 were recruited from the primary school “Marymount Institute” of Rome (Italy). Following baseline assessment, we randomly classes assigned to one of the three conditions: creativity ABs (CREAT; *n* = 40), fitness ABs (FIT; *n* = 51), and control (CON; *n* = 50) on the basis of the type of protocol performed three times in the same school day. Participants’ demographics are presented in Table 1. The only exclusion criterium was to be free from injury, which could preclude the PA practice. The school principal, teachers, and parents of the involved classes were provided with an information statement and were informed regarding the aim of the study. All students received the programs as a whole-class intervention. The parents of the involved children signed the written consent form. The study was designed and conducted in accordance with the Declaration of Helsinki and approved by the bioethical local committee of University of Rome “Foro Italico” (University Committee for Research (CAR-IRB), Code: 61/2020).

### 2.3. Procedures

To assess attentive and academic performances, we administered the Stroop Color and Word Test (SCWT), Victoria version [35], and the Math (MATH) test [36]. In addition, the degree of enjoyment was evaluated for each group through the modified Physical Activity Enjoyment Scale (PACES) [37]. All the students were evaluated ad the same time and at the same day.

#### 2.3.1. Math Test

Mathematics performances were measured using a stage-appropriate version of MATH [36]. This test included basic calculations, problems, magnitude comparison tasks, and number ordering tasks. The test was collectively administered, and participants were required to complete as many math tasks as possible in 30 min. The total score was obtained, summing the numbers of correct responses in each area (ICC 0.95) [38]. The test requirements were age-related and structured according to the core curriculum of primary schools. This test has a test–retest reliability of 0.70 [36].

#### 2.3.2. Stroop Color and Word Test

The Stroop Color and Word Test (SCWT) consists of three cards, with six rows of four items. The first section (C) contains 24 rectangles printed in four colors (blue, green, red, or yellow); the subject has to recognize the color as quick as possible. The second section (W) contains 24 common words printed in the four colors; the subject has to name the color, ignoring the written word. The third section (CW) contains 24 color name words printed in an incongruent color (e.g., word blue printed in red; in this case, the subject must name the word “red”). The number of errors and the time spent to complete each condition were recorded. The time interference score (TI) and the error interference score (EI) were calculated by adopting the method proposed by Caffarra [39] and using the following formulas:

TI = CWT − [(WT + CT)/2]

where TI: time interference score; WT: time to complete W condition; CT: time to complete C condition; CWT: time to complete CW condition.

EI = CWE − [(WE + CE)/2]

where EI: error interference score; EI: errors interference score; WE: errors in W condition; CE: errors in C condition; CWE: errors in CW condition.

This test has a good test–retest reliability (0.86 for the W score, 0.82 for the C score, 0.73 for the CW score) [40].

#### 2.3.3. Paces

To assess the subjective degree of enjoyment during the ABs, we used the Physical Activity Enjoyment Scale (PACES). The PACES, validated for the Italian version [37], has been modified in a simplify version for young aged. This test was composed of a 5-item Likert scale. A total score of 10 or less indicated a low enjoyment, a score between 11 and 15 indicated a medium enjoyment, and a score between 16 and 20 indicated a high enjoyment of PA. The questionnaire has a good test–retest reliability, with Cronbach alpha values ranging from 0.78 and 0.89.

### 2.4. Intervention

The PA intervention was organized into three exercise sessions (ABs) scheduled during the same school day. Each session lasted 15 min.

Each class was randomly assigned to the three different ABs protocols: CREAT, FIT, and CON. The activities were carried out by MCs students of University of Rome “Foro Italico”.

The CREAT was based on the combination of cognitive–creative and conditional tasks, such as improvisations, dramatization of events or brief stories, and simulation and imitation games, that arouse the creative process and improve the expression of emotions, in interaction with the environmental constraints. The constrains, used by the teacher, limit the degrees of freedom (e.g., rules, spatial position of the partner, velocity of execution), stimulating new solutions to the tasks’ realization.

The FIT protocol was designed to propose moderate to vigorous PA including strength and aerobic activities such as squat, jumping jacks, lunges, and running on the spot. Children were asked to imitate the teacher movements with not much time left for explanation. The two ABs were proposed with musical accompaniment.

In the control condition, children remained seated and were involved in social interactions with the research staff.

### 2.5. Statistical Analysis

For all statistical tests, Statistica package (Statsoft v.10, StatSoft Inc., Oklahoma, OK, USA) was used, and the significance level was set to *α* = 0.05.

All variables were tested for normal distribution via the Shapiro–Wilk test. A factorial analysis of variance (ANOVA) with group (CREAT, FIT, CON), class (third, fourth, fifth), enjoyment (high, medium, low), and sex (males, females) as between factors and time of the assessment (pre, post) as a repeated factor was employed to test the acute effects of active rests on the MATH, on time (SCWT-time) and errors (SWCT-error). The level of enjoyment expressed by the participants was tested using a three-way ANOVA with group, class, and sex as between factors.

Main effects and interactions were further analyzed using the Tukey post hoc test. Achieved statistical power (*β*) and the effect size (*_p_η*^2^ and Cohen’s *d*) were reported and interpreted as suggested by Cohen [41].

## 3. Results

**Figure 1:**
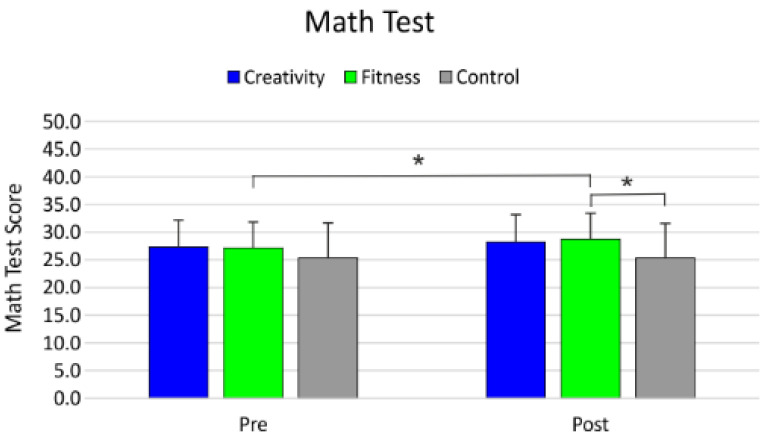
Math test results. * significant differences.

A significant main effect of group on math test (F_2,123_ = 3.68; *p* = 0.027; β = 0.66; *_p_η*^2^ = 0.06) was found. The Tukey post hoc test revealed that FIT had better performance than CON (*p* = 0.023; d = 0.597), whereas no difference emerged between CREAT and FIT and between CREAT and CON (all *p*s > 0.05). A significant main effect of time of assessment emerged for the math test (F_1,123_ = 10.82; *p* = 0.001; β = 0.90; *_p_η*^2^ = 0.08). The post hoc test revealed larger scores in the post intervention assessment (pre: 26.6, post: 27.3; *p* < 0.001; d = 0.155). A significant group by time interaction was observed for the math test (F_2,123_ = 5.40; *p* = 0.005; β = 0.83; *_p_η*^2^ = 0.08), and the Tukey post hoc test showed that FIT improved its performance in the math test after the intervention (FIT pre: 27.1; FIT post: 28.8; *p* < 0.001; d = 0.344), with better performance than CON (FIT post: 28.8; CON post: 25.3; *p* = 0.011; d = 0.621) (Figure 1, Table 2).

**Figure 2:**
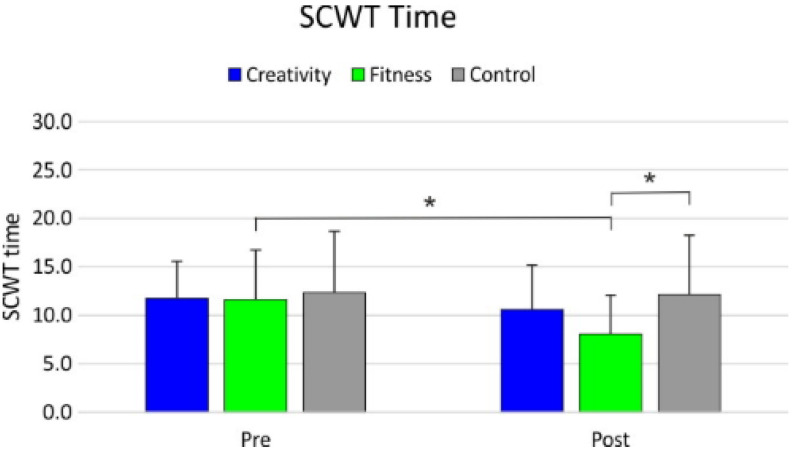
SCWT (time) results. * significant differences.

As regards the SCWT time, the factorial ANOVA revealed a significant effect of group (F_2,123_ = 3.12; *p* = 0.047; β = 0.59; *_p_η*^2^ = 0.048), with FIT being faster than CON (9.83 vs. 12.23; *p* = 0.034; d = 0.428). No difference in the SCWT time was observed between FIT and CREAT (9.83 vs. 11.00; *p* = 0.48) and between CREAT and CON (*p* = 0.45). In addition, a significant main effect of time of assessment was observed (F_1,123_ = 18.83; *p* < 0.001; β = 0.99; *_p_η*^2^ = 0.13), with the post-intervention assessment showing faster execution (pre: 11.8) than the pre-intervention assessment (post: 10.2; *p* < 0.001). The factorial ANOVA also revealed a significant group by time interaction (F_2,123_ = 10.47; *p* < 0.001; β = 0.98; *_p_η*^2^ = 0.14). The post hoc analysis revealed an enhanced performance in the FIT in the post-intervention assessment compared to the pre-intervention assessment (pre: 11.6; post: 8.1; *p* < 0.001; d = 0.764). FIT showed faster performance in the post-intervention assessment than CON (FIT: 8.1; CON: 12.1; *p* = 0.002; d = 0.787) (Figure 2, Table 3).

**Figure 3:**
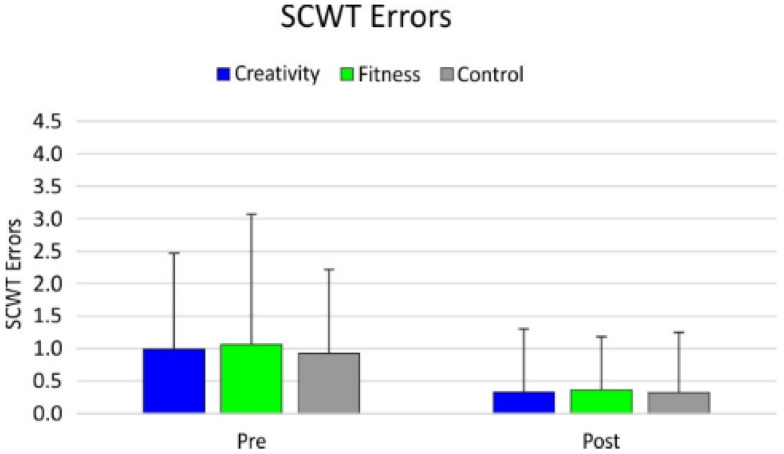
SCWT (errors) results.

The factorial ANOVA showed a main effect of time of assessment for the SCWT errors (F_1,123_ = 17.27; *p* <0.001; β = 0.98; *_p_η*^2^ = 0.12), with the post-intervention assessment showing more accurate performance than the pre-intervention assessment (pre: 0.99; post: 0.34; *p* < 0.001; d = 0.496). No significant difference amongst groups was found (Figure 3).

**Figure 4:**
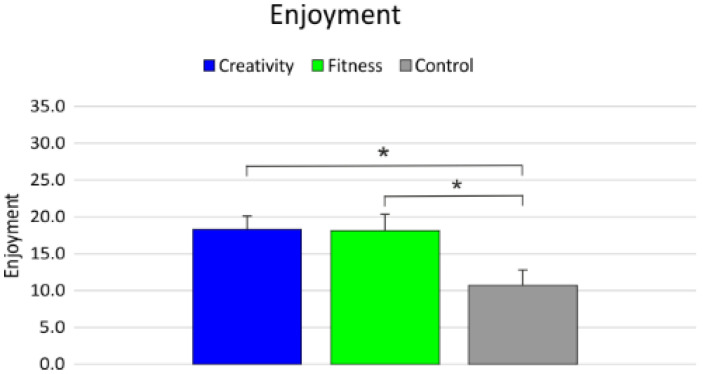
Degree of enjoyment. * significant differences.

The statistical analysis on the level of enjoyment showed a significant main effect of group (F_2,123_ = 178.46; *p* < 0.001; β = 0.99; *_p_η*^2^ = 0.74). The post hoc analysis showed that both CREAT (18.3) and FIT (18.2) had larger enjoyment scores than CON (10.7; all *p* < 0.001; d = 3.83, d = 3.46, respectively, for CREAT and FIT) (Figure 4, Table 4).

## 4. Discussion

This study aimed to find out the immediate influence of different types of ABs, repeated three times in the same school day, on attentive skills and mathematical performance.

Despite previous studies reporting small acute effects on academic performance following ABs [9,42], the participants of this study showed significant positive results between pre- and post-intervention on attention levels and MATH tasks. Acute PA generates a release of neurotransmitters that enhance arousal and attention, improving learning functions and consequently mathematical performance [2].

Considering all the participants as one group, we found significant improvement in SCWT time and errors. Yanagisawa [43] showed that an acute bout of moderate exercise enhanced the Stroop interference activation. A lasting learning effect might have partially influenced these test–retest results, due to the repetitive administration of the Stroop test with an interval of only a week. Practice effect on Stroop performance has been well documented by different studies [44,45,46]. Nevertheless, when we divided the sample into the three groups, only FIT significantly improved the SCWT time of execution from pre- to post-assessment. No significant differences were found between the two experimental protocols, while the FIT achieved better results than CON. Acute cardiovascular exercise increases cerebral oxygenation in the pre-frontal cortex [47].

Considering the math test, to avoid the learning effect, we changed the procedures and values of the math test from the first to the second administration. Only FIT significantly improved the math performance from pre- to post-assessment. Comparing the two experimental protocols, we found no significant differences, while only FIT showed significantly better results than CON. It has been shown that single bouts of PA (acute exercise) cause physiological arousal, facilitating the available attentional resources and the engagement of cognitive functioning [48]. It is possible that higher PA intensity levels in the cardiovascular condition contributed to higher scores. The cardiovascular protocol used music to provide the executive rhythm (music beats), and the work/rest ratio was correctly organized by the teacher. These conditions allowed children to reach a good level of exertion. It was previously shown that lasting fitness and cognitive effects, especially on attention, were obtained after more intense exercise intervention [49]. The rhythmic pumping of the hearth, in the concurrence between tempo of music and exercise, generates a similar resonant frequency in the brain, influencing the autonomic nervous system [50]. The use a synchronous rhythm between music and exercise could be the explanation for the cognitive functions’ improvement [28]. In the creative protocol, the activity started from an event-telling and its dramatization, in order to promote the transition from the children’s imaginative skill to the deliberate PA, giving the children support to try. In this protocol, the music was used only to elicit emotional experiences and positive affective regulation, since cognitive creativity is enhanced by listening to music during exercise [51]. As Runco [52] showed, creativity stimulates the divergent thinking, requiring finding new ideas in motor problem solving tasks more than promotion of high exertion. It was hypothesized that, despite these recognized benefits of creative tasks, the acute bout of this protocol probably did not reach the optimal intensity to promote better acute effects, such as the cardiovascular protocol. The time needed by the teachers to explain to the children the tasks and the execution modalities could have reduced the effective exertion time.

PA, especially that performed with music, is closely linked with enjoyment. Enjoyment is a fundamental factor in the learning process, helping children to better retain information and develop new knowledge [34], as well as showing a positive correlation with students’ use of cognitive learning strategies [53,54]. No significant difference level of enjoyment was found between the two experimental protocols, confirming that both of the types of ABs were appreciated by the children. The FIT and CREAT outperformed the control group.

### Limitations

For organizational needs, this study was a class-level intervention.

The exercise intensity was assessed with Omni-Scale method; however, participants were too young to be able to identify their individual RPE. Therefore, this assessment variable was not considered in this paper.

Since the ABs were simultaneously performed, the outcomes could be operator-dependent.

The music was used in both the experimental ABs. Nevertheless, the influence of this variable on math performance and attention was not assessed.

The brief interval between pre- and post-test administration could have not avoided a possible learning effect in the Stroop Color and Word test that could have altered the results of AB effects on attention.

## 5. Conclusions

COVID-19 restrictions prevented children from achieving recommended levels of PA [55]. School reopening allowed children to have access to school-based physical activities; however, its educational programs will optimize time and resources. A new plan and changes in timetables, implementing PA during the school-time, may insure children are more physically active.

This study showed how PA interventions can positively affect attention and academic performance in children [56,57].

These effects are more pronounced when these interventions enhance children’s motivation and higher level of enjoyment [54]. The cardiovascular approach provides the highest results in term of short-term efficacy; the better time on/off of the cardiovascular protocol may have allowed the proper exercise volume (duration × intensity). Nevertheless, it is possible that cardiovascular protocol, reply in a chronic administration, may lead to a loss of motivational impact or enjoyment. In contrast, the creative protocol may provide new stimuli and motivation improvements, since it provides a major engagement on cognitive performance. This report provided useful information for teachers to organize future active breaks between curricular lessons. As guidelines, an active break plane based on the turnover of creative and cardiovascular active break protocols in the same school day could be proposed. School in the present time represents the only one pillar to comply with the current WHO guidelines on the minimum PA dose per day for children. Promoting adequate levels of PA in children is a major public health issue.

## Figures and Tables

**Table 1:** Sample characteristics.

Variable	*n*
**Total**	141
**Age (Mean ± SD)**	9.61 ± 0.82
Creativity	9.58 ± 0.85
Fitness	9.57 ± 0.82
Control	9.66 ± 0.82
**Creativity**	40
Male	14
Female	26
Third grade	11
Fourth grade	18
Fifth grade	11
**Fitness**	51
Male	26
Female	25
Third grade	15
Fourth grade	19
Fifth grade	17
**Control**	50
Male	28
Female	22
Third grade	14
Fourth grade	17
Fifth grade	19

**Table 2:** Math test results.

Variable	F	*p*	*_p_η* ^2^	β
Sex	3.60	0.060	0.028	0.469
Group	3.69	0.028	0.057	0.668
Class	2.47	0.089	0.039	0.488
Sex by group	2.59	0.079	0.040	0.509
Sex by class	1.08	0.343	0.017	0.236
Group by class	1.41	0.236	0.044	0.427
Time	10.82	0.001	0.081	0.904
Time by sex	3.45	0.066	0.027	0.454
Time by group	5.40	0.006	0.081	0.837
Time by class	2.44	0.091	0.038	0.484

**Table 3:** SCWT test results.

Variable	F	*p*	*_p_η* ^2^	β
Sex	0.6	0.457	0.005	0.115
Group	3.1	0.050	0.047	0.582
Class	3.4	0.037	0.052	0.630
Sex by group	0.3	0.715	0.005	0.103
Sex by class	1.6	0.203	0.026	0.336
Group by class	0.2	0.952	0.006	0.085
Time	84.4	0.000	0.407	1.000
Time by sex	0.0	0.897	0.000	0.052
Time by group	18.4	0.000	0.230	1.000
Time by class	1.5	0.220	0.024	0.321

**Table 4:** Degree of enjoyment results.

Variable	F	*p*	*_p_η* ^2^	β
Sex	0.2	0.643	0.002	0.075
Group	178.5	0.000	0.744	1.000
Class	4.2	0.017	0.064	0.730
Sex by group	0.2	0.848	0.003	0.075
Sex by class	0.8	0.463	0.012	0.179
Group by class	0.5	0.752	0.015	0.160

## Data Availability

Data that support the findings of this study are available from the corresponding author upon reasonable request.
